# Supplementary material for: High-Density Lipoprotein Suppresses Neutrophil Extracellular Traps Enhanced by Oxidized Low-Density Lipoprotein or Oxidized Phospholipids
Source: Int J Mol Sci. 2022 Nov 13;23(22):13992. doi: 10.3390/ijms232213992 (PMC9698465; doi:10.3390/ijms232213992)
Supplement: Supplementary file 1 [file ijms-23-13992-s001.zip › ijms-1985050-supplementary.pptx]

## Slide 1
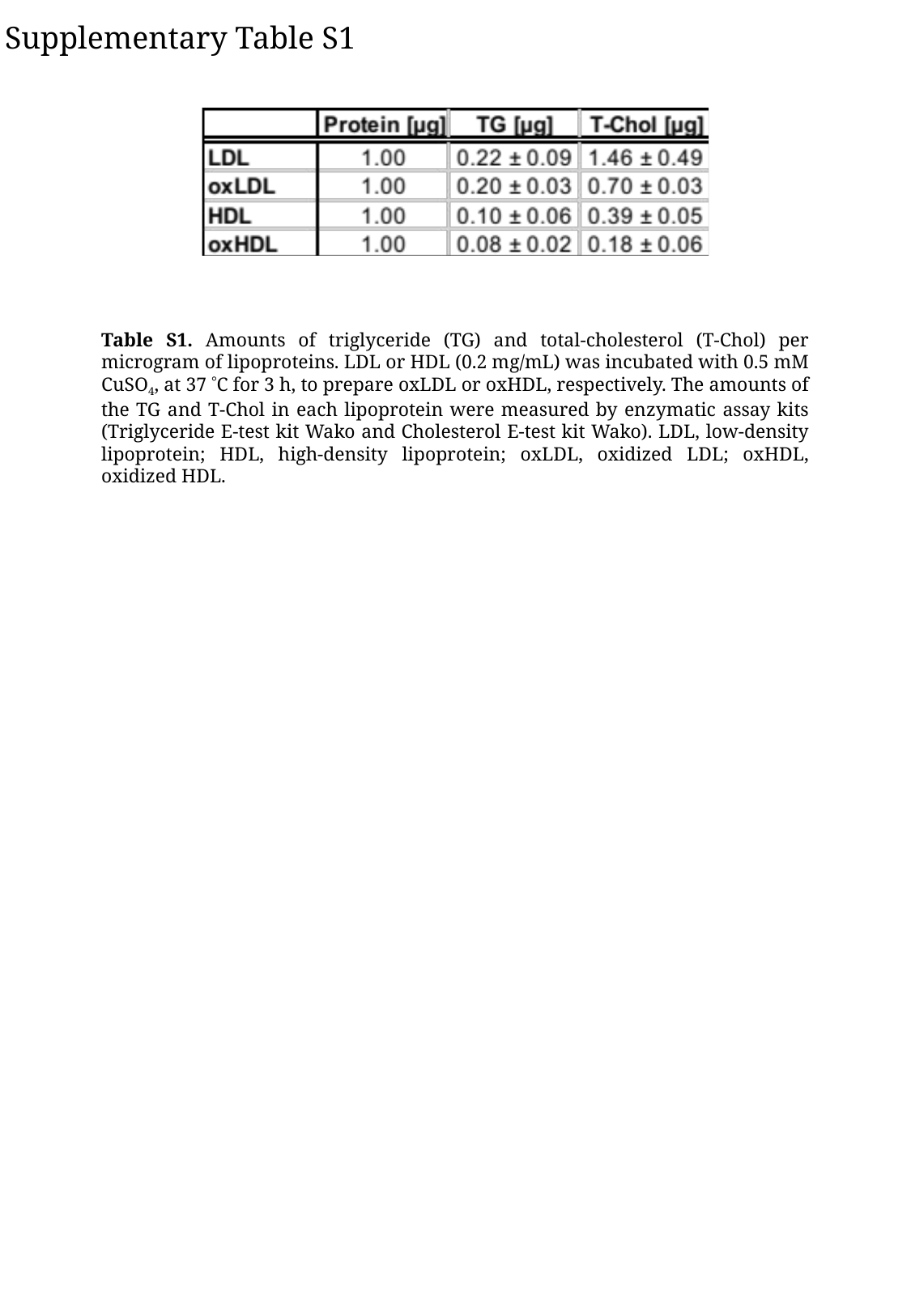

Supplementary Table S1
Table S1. Amounts of triglyceride (TG) and total-cholesterol (T-Chol) per microgram of lipoproteins. LDL or HDL (0.2 mg/mL) was incubated with 0.5 mM CuSO4, at 37 °C for 3 h, to prepare oxLDL or oxHDL, respectively. The amounts of the TG and T-Chol in each lipoprotein were measured by enzymatic assay kits (Triglyceride E-test kit Wako and Cholesterol E-test kit Wako). LDL, low-density lipoprotein; HDL, high-density lipoprotein; oxLDL, oxidized LDL; oxHDL, oxidized HDL.

## Slide 2
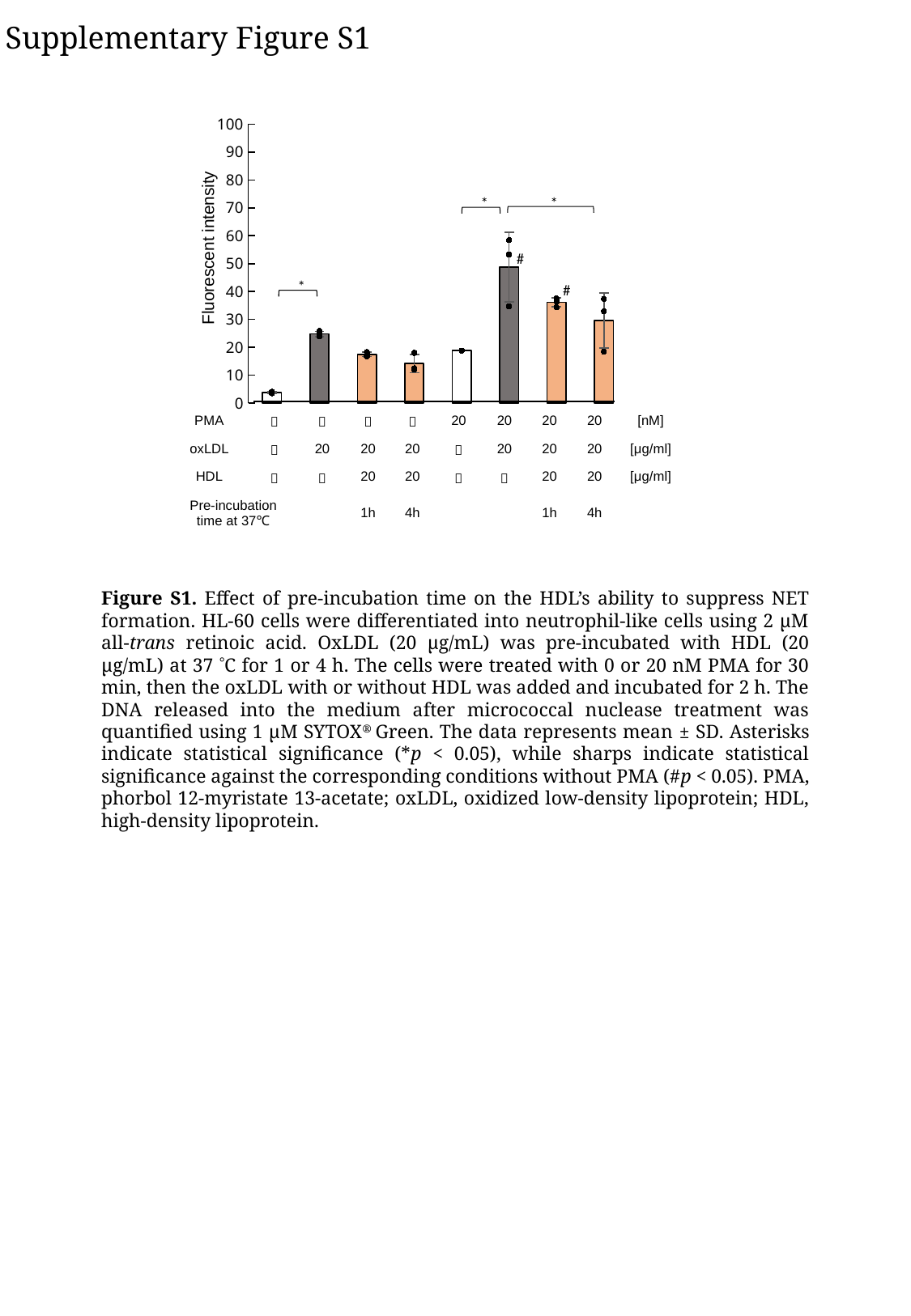

Supplementary Figure S1
### Chart
| Category | A.V. | #1 | #2 | #3 |
|---|---|---|---|---|
| PBS/EDTA | 3.834635 | 3.9343150000000002 | 3.504025 | 4.065564999999999 |
| oxLDL20 | 24.76293166666667 | 24.490865000000003 | 23.975165 | 25.822765 |
| 1h oxLDL20/HDL20 | 17.427198333333333 | 16.699065 | 17.319865 | 18.262665000000002 |
| 4h oxLDL20/HDL20 | 14.156565 | 17.990065 | 12.014764999999999 | 12.464865 |
| PBS/EDTA | 18.820965 | None | 18.820965 | None |
| oxLDL20 | 48.789031666666666 | 58.401264999999995 | 53.280764999999995 | 34.685064999999994 |
| 1h oxLDL20/HDL20 | 36.09119833333333 | 36.396964999999994 | 37.529665 | 34.346965 |
| 4h oxLDL20/HDL20 | 29.573665000000002 | 37.362465 | 32.922264999999996 | 18.436265000000002 |
*
*
Fluorescent intensity
#
*
#
| PMA | ー | ー | ー | ー | 20 | 20 | 20 | 20 | [nM] |
| --- | --- | --- | --- | --- | --- | --- | --- | --- | --- |
| oxLDL | ー | 20 | 20 | 20 | ー | 20 | 20 | 20 | [μg/ml] |
| HDL | ー | ー | 20 | 20 | ー | ー | 20 | 20 | [μg/ml] |
| Pre-incubation time at 37℃ | | | 1h | 4h | | | 1h | 4h | |
Figure S1. Effect of pre-incubation time on the HDL’s ability to suppress NET formation. HL-60 cells were differentiated into neutrophil-like cells using 2 μM all-trans retinoic acid. OxLDL (20 μg/mL) was pre-incubated with HDL (20 μg/mL) at 37 °C for 1 or 4 h. The cells were treated with 0 or 20 nM PMA for 30 min, then the oxLDL with or without HDL was added and incubated for 2 h. The DNA released into the medium after micrococcal nuclease treatment was quantified using 1 μM SYTOX®︎ Green. The data represents mean ± SD. Asterisks indicate statistical significance (*p < 0.05), while sharps indicate statistical significance against the corresponding conditions without PMA (#p < 0.05). PMA, phorbol 12-myristate 13-acetate; oxLDL, oxidized low-density lipoprotein; HDL, high-density lipoprotein.

## Slide 3
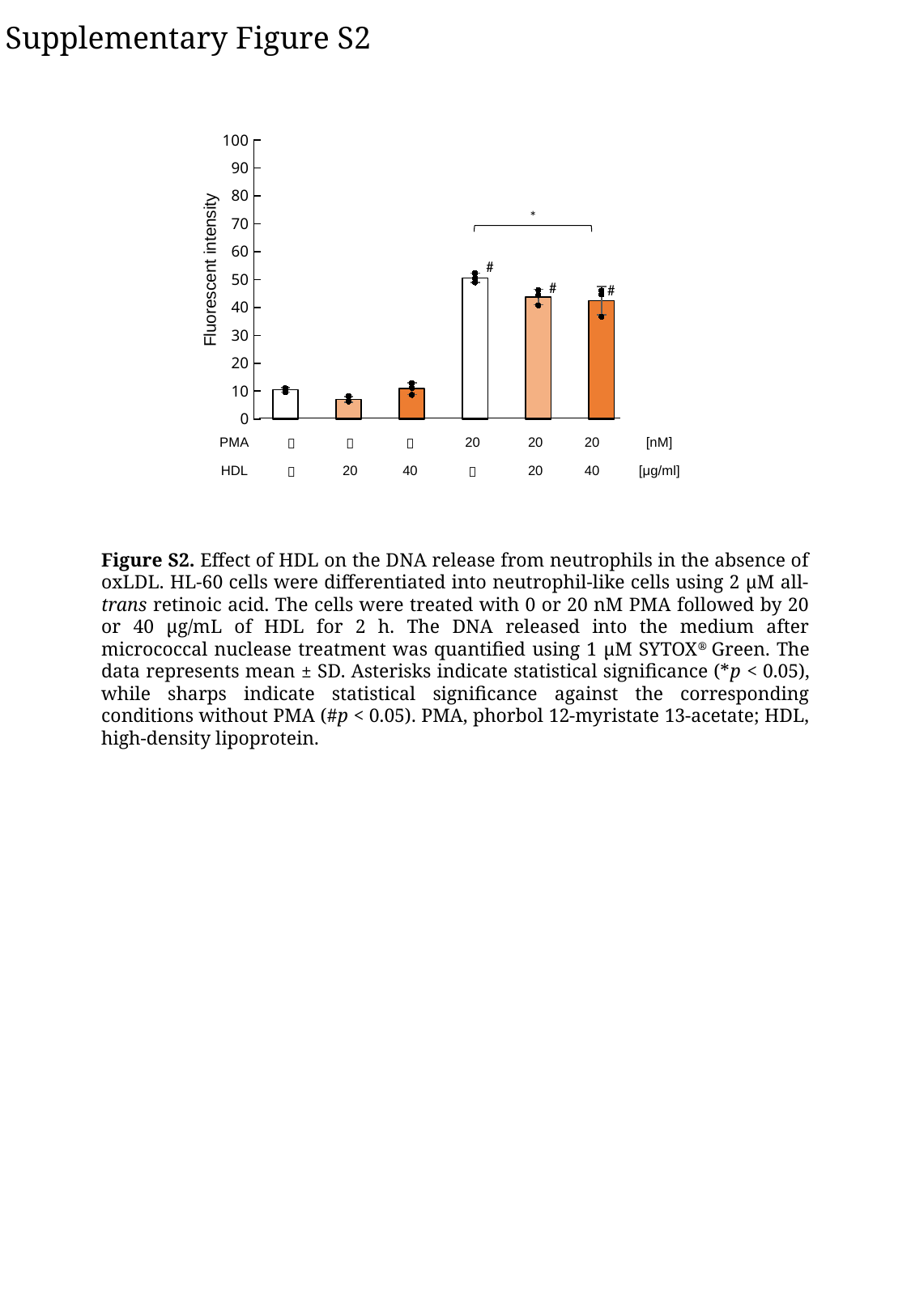

Supplementary Figure S2
### Chart
| Category | AV | #1 | #2 | #3 |
|---|---|---|---|---|
| PBS | 10.441138 | 10.693978 | 11.036578 | 9.592858 |
| HDL20 | 7.001767999999999 | 6.310188 | 8.153448 | 6.541668 |
| HDL40 | 10.861951333333332 | 12.789378 | 11.153578 | 8.642897999999999 |
| PBS | 50.60674466666668 | 50.525578 | 52.324078 | 48.970578 |
| HDL20 | 43.75904466666666 | 46.183578 | 40.756078 | 44.337478 |
| HDL40 | 42.444211333333335 | 36.677878 | 44.648778 | 46.005978 |
*
#
Fluorescent intensity
#
#
| PMA | ー | ー | ー | 20 | 20 | 20 | [nM] |
| --- | --- | --- | --- | --- | --- | --- | --- |
| HDL | ー | 20 | 40 | ー | 20 | 40 | [μg/ml] |
Figure S2. Effect of HDL on the DNA release from neutrophils in the absence of oxLDL. HL-60 cells were differentiated into neutrophil-like cells using 2 μM all-trans retinoic acid. The cells were treated with 0 or 20 nM PMA followed by 20 or 40 μg/mL of HDL for 2 h. The DNA released into the medium after micrococcal nuclease treatment was quantified using 1 μM SYTOX®︎ Green. The data represents mean ± SD. Asterisks indicate statistical significance (*p < 0.05), while sharps indicate statistical significance against the corresponding conditions without PMA (#p < 0.05). PMA, phorbol 12-myristate 13-acetate; HDL, high-density lipoprotein.

## Slide 4
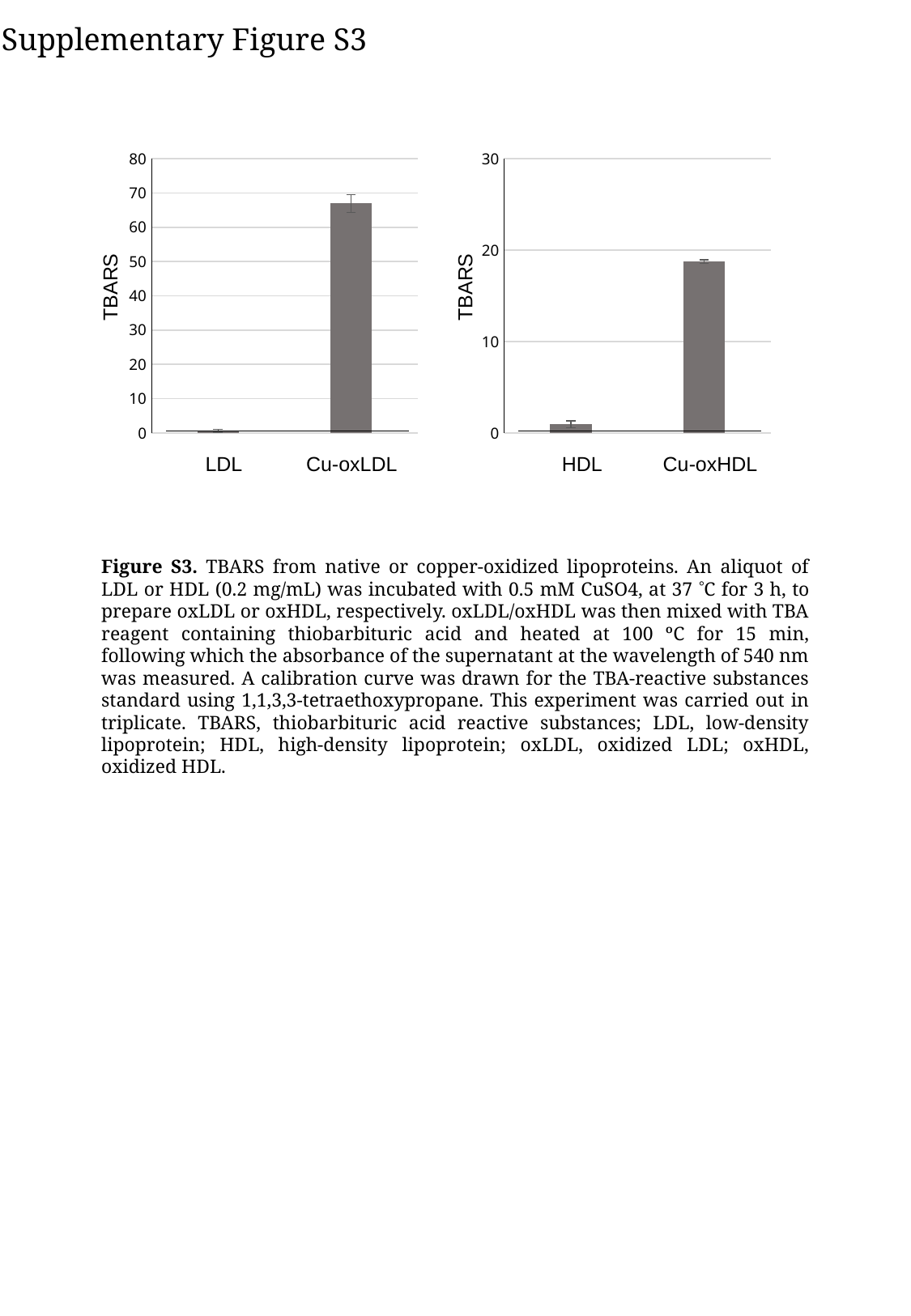

Supplementary Figure S3
### Chart
| Category | TBARS |
|---|---|
| HDL | 0.9585430146177807 |
| Cu-oxHDL | 18.751497723460343 |
### Chart
| Category | TBARS |
|---|---|
| LDL | 0.6589983225497237 |
| Cu-oxLDL | 66.91828420800383 |TBARS
TBARS
LDL
Cu-oxLDL
HDL
Cu-oxHDL
Figure S3. TBARS from native or copper-oxidized lipoproteins. An aliquot of LDL or HDL (0.2 mg/mL) was incubated with 0.5 mM CuSO4, at 37 °C for 3 h, to prepare oxLDL or oxHDL, respectively. oxLDL/oxHDL was then mixed with TBA reagent containing thiobarbituric acid and heated at 100 ºC for 15 min, following which the absorbance of the supernatant at the wavelength of 540 nm was measured. A calibration curve was drawn for the TBA-reactive substances standard using 1,1,3,3-tetraethoxypropane. This experiment was carried out in triplicate. TBARS, thiobarbituric acid reactive substances; LDL, low-density lipoprotein; HDL, high-density lipoprotein; oxLDL, oxidized LDL; oxHDL, oxidized HDL.

## Slide 5
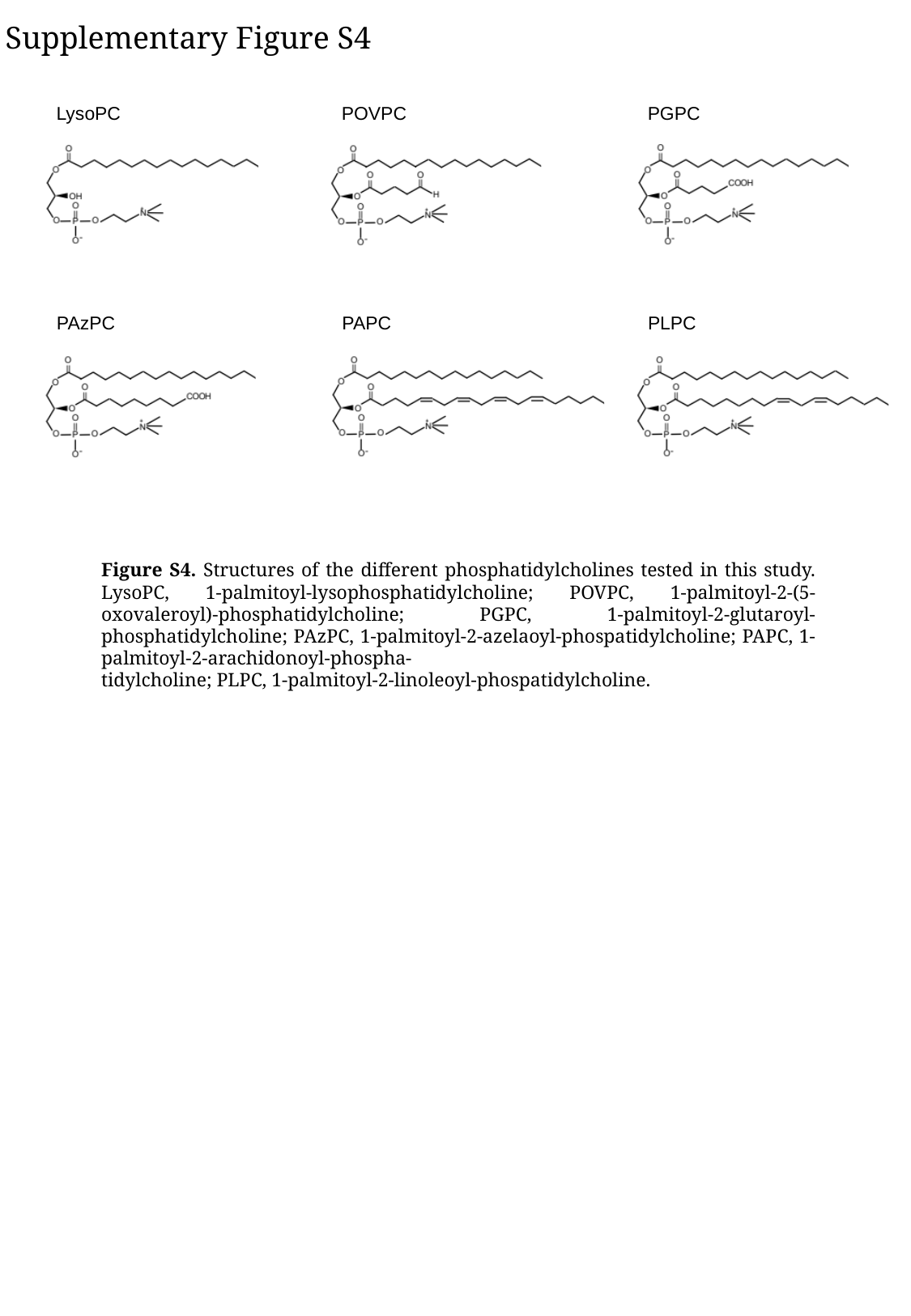

Supplementary Figure S4
LysoPC
POVPC
PGPC
PAzPC
PAPC
PLPC
Figure S4. Structures of the different phosphatidylcholines tested in this study. LysoPC, 1-palmitoyl-lysophosphatidylcholine; POVPC, 1-palmitoyl-2-(5-oxovaleroyl)-phosphatidylcholine; PGPC, 1-palmitoyl-2-glutaroyl-phosphatidylcholine; PAzPC, 1-palmitoyl-2-azelaoyl-phospatidylcholine; PAPC, 1-palmitoyl-2-arachidonoyl-phospha-
tidylcholine; PLPC, 1-palmitoyl-2-linoleoyl-phospatidylcholine.

## Slide 6
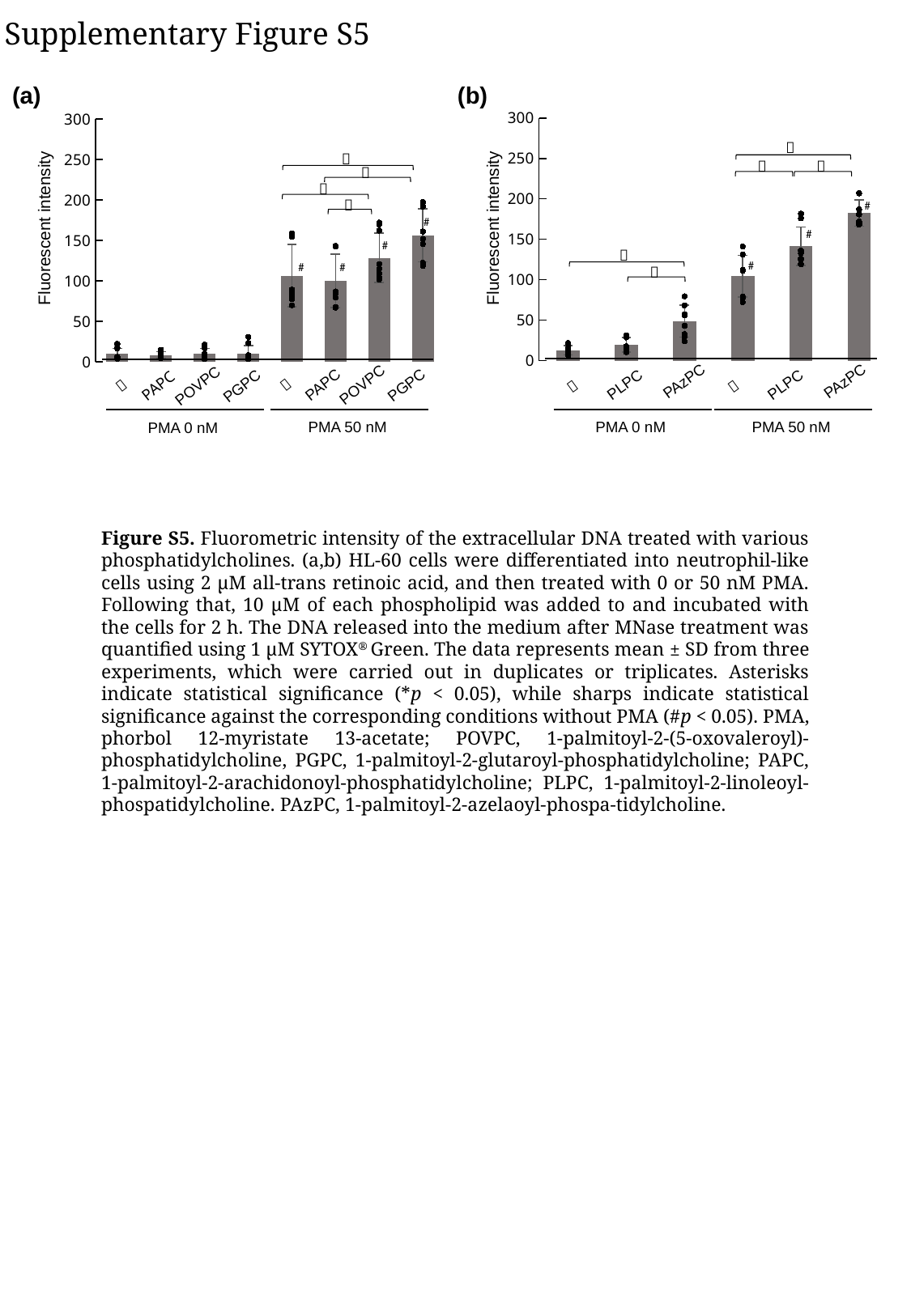

Supplementary Figure S5
(a)
(b)
### Chart
| Category | AV | #1 | #2 | #3 | #4 | #5 | #6 | #7 | #8 |
|---|---|---|---|---|---|---|---|---|---|
| RPMI | 12.841129250000002 | 8.4207965 | 6.5805565 | 6.4813665 | 11.773908500000001 | 20.6557085 | 11.3749085 | 15.8450945 | 21.5966945 |
| PLPC | 19.57703175 | 17.8170465 | 15.257346499999999 | 11.826646499999999 | 31.3827085 | 28.7109085 | 30.7369085 | 10.4211945 | 10.4634945 |
| PAzPC | 48.76989424999999 | 29.503846499999998 | 23.8415465 | 43.1412465 | 68.3662085 | 55.8767085 | 57.0761085 | 32.8234945 | 79.5299945 |
| RPMI | 104.45541924999998 | 79.2876465 | 77.0830465 | 72.2461465 | 112.2695085 | 111.06550849999999 | 111.4215085 | 141.1054945 | 131.1644945 |
| PLPC | 141.54023175 | 126.2094465 | 124.8784465 | 119.02344649999999 | 132.82850850000003 | 136.0435085 | 135.6405085 | 175.9744945 | 181.72349450000002 |
| PAzPC | 182.71260675000002 | 180.8214465 | 172.0734465 | 187.05444649999998 | 169.3895085 | 168.08950850000002 | 170.4905085 | 206.9064945 | 206.8754945 |
### Chart
| Category | AV | #1 | #2 | #3 | #4 | #5 | #6 | #7 | #8 | #9 |
|---|---|---|---|---|---|---|---|---|---|---|
| RPMI | 9.590047444444444 | 5.9466975 | 4.7405775000000006 | 3.8695075 | 22.0821445 | 16.4309445 | 17.4447445 | 4.865327000000001 | 4.9454970000000005 | 5.984987 |
| PAPC | 8.145926333333334 | 8.503467500000001 | 5.6007275000000005 | 5.0699975 | 11.6492445 | 14.881944500000001 | 14.0662445 | 4.886277000000001 | 4.333617 | 4.321817 |
| POVPC | 9.936683 | 5.3508575 | 9.6392375 | 9.3199475 | 21.0546445 | 16.696844499999997 | 16.7509445 | 3.551317 | 3.7721470000000004 | 3.294207 |
| PGPC | 9.782500777777777 | 4.5291975 | 4.1868375 | 3.9561875000000004 | 30.721344499999997 | 23.097944499999997 | 8.208944500000001 | 5.5768770000000005 | 4.170257 | 3.5949169999999997 |
| RPMI | 106.41130522222221 | 69.7926375 | 81.3359375 | 82.6064375 | 157.88404450000002 | 158.75904450000002 | 154.6070445 | 86.25096699999999 | 77.207567 | 89.258067 |
| PAPC | 99.932983 | 67.4999375 | 79.2803375 | 66.9808375 | 143.2020445 | 142.58104450000002 | 142.9660445 | 86.924667 | 85.85496699999999 | 84.106967 |
| POVPC | 128.5297607777778 | 109.1917375 | 114.7607375 | 120.6837375 | 172.09304450000002 | 169.8230445 | 162.2560445 | 104.392167 | 101.897167 | 101.67016699999999 |
| PGPC | 156.32031633333332 | 145.6067375 | 151.7637375 | 161.0537375 | 197.4340445 | 191.85404450000001 | 197.1800445 | 122.382167 | 120.962167 | 118.64616699999999 |＊
＊
＊
＊
＊
＊
＊
#
#
Fluorescent intensity
Fluorescent intensity
#
#
＊
#
#
#
＊
PAzPC
PAzPC
ー
POVPC
PLPC
PLPC
PAPC
PGPC
ー
POVPC
PAPC
PGPC
ー
ー
PMA 50 nM
PMA 0 nM
PMA 50 nM
PMA 0 nM
Figure S5. Fluorometric intensity of the extracellular DNA treated with various phosphatidylcholines. (a,b) HL-60 cells were differentiated into neutrophil-like cells using 2 μM all-trans retinoic acid, and then treated with 0 or 50 nM PMA. Following that, 10 μM of each phospholipid was added to and incubated with the cells for 2 h. The DNA released into the medium after MNase treatment was quantified using 1 μM SYTOX®︎ Green. The data represents mean ± SD from three experiments, which were carried out in duplicates or triplicates. Asterisks indicate statistical significance (*p < 0.05), while sharps indicate statistical significance against the corresponding conditions without PMA (#p < 0.05). PMA, phorbol 12-myristate 13-acetate; POVPC, 1-palmitoyl-2-(5-oxovaleroyl)-phosphatidylcholine, PGPC, 1-palmitoyl-2-glutaroyl-phosphatidylcholine; PAPC, 1-palmitoyl-2-arachidonoyl-phosphatidylcholine; PLPC, 1-palmitoyl-2-linoleoyl-phospatidylcholine. PAzPC, 1-palmitoyl-2-azelaoyl-phospa-tidylcholine.
